# Creating online cognitive assessment platform for children’s literacy and math skills: a case study illustrated with the ACE kids platform

**DOI:** 10.3389/fnhum.2026.1882555

**Published:** 2026-10-08

**Authors:** V. S. H. Ngan, J. Mo, O. K. P. Wong, M. C. Y. Ng, J. C. S. Ho, T. Inoue, C. McBride, U. Maurer

**Affiliations:** 1 Department of Psychology, The Chinese University of Hong Kong, Hong Kong, Hong Kong SAR, China; 2 School of Education, University of Saint Joseph, Macau, Macao SAR, China; 3 Centre for Developmental Psychology, The Chinese University of Hong Kong, Hong Kong, Hong Kong SAR, China; 4 Department of Human Development and Family Science, Purdue University, West Lafayette, IN, United States; 5 Brain and Mind Institute, The Chinese University of Hong Kong, Hong Kong, Hong Kong SAR, China

**Keywords:** child assessment, computer-assisted testing, gamification, online assessment, remote assessment

## Abstract

Computerized versions of paper-and-pencil tests made behavioral assessments of children more automatic and precise. The COVID-19 pandemic further demonstrated the need for assessments to be offered online to allow for remote data collection. However, the design and implementation of computerized tests in an online environment come with various challenges. This article probes the issues, needs and challenges for the development of online assessment of reading and maths, and related cognitive abilities. The paper aims to inform researchers about logistical and technical considerations of developing a customized platform for online child data collection, including (a) technical considerations, (b) interface design, (c) data management, and (d) planning for validation against paper-and-pencil measures. These issues are illustrated with experiences gleaned from the development of an online assessment platform (ACE Kids platform), designed to deliver over 40 tests and questionnaires related to Chinese and English literacy as well as mathematics abilities in K3 – P6 children. The paper also highlights the unique challenges surrounding building online, self-directed biliteracy assessment involving a large assessment battery. Furthermore, we discuss features that improve the ease of use, instructional clarity, and retention of attention. We discussed the use of questionnaires to sample child usage experience during early stages of assessment development to optimize design. Finally, we give consolidated advice for the planning, development and data collection stages for such a customized assessment platform.

## 1 Introduction

Assessing learning and development for children is critical for tracking progress and milestones, essential for research and theory building, as well as for clinical purposes (Mash and Hunsley, 2005; Van Hemel and Snow, 2008). Traditionally, assessment has been done in supervised, paper-and-pencil sessions, in which trained proctors deliver test materials and record responses. Nonetheless, advances in digital technology and improvement in internet speed and access have made large-scale technology-assisted child assessment possible. A computerized mode of delivery fits well with modern media consumption of children, who have also increasingly grown familiar with operating computers and tablets (Clemente-Suárez et al., 2024; Kabali et al., 2015). In the US, 82% of 12th graders visited social media every day while only 16% read a printed book or magazine in 2016, and this followed a 40-year trend of decline of print reading and increase in on-screen reading (Twenge et al., 2019), a trend that continued on subsequently (Peras et al., 2023). The COVID-19 pandemic also necessitated remote data collection and the adoption of online educational aids in schools (Greenhow et al., 2021), further increasing screen time (Ribner et al., 2021).

A digital mode of assessment of child reading and cognitive abilities carries considerations that are unique and different from traditional assessments. First, there is the difference in medium, from assessments delivered using paper and pencil, to computerized assessment leveraging electronic devices with screens and varied input methods (mouse, keyboard, touchscreen, drawing tablet, microphone, even camera capable of capturing eye movements); the diversity of modality creates potential differences in data (Dadey et al., 2018). Second, assessments can be hosted online and accessed both from local (research labs or schools) and remote locations (homes or any other spaces), the variability of which is a source of ease of access and uptake, but also of distractions and noises in data (Vinas-Guasch et al., 2023). Third, assessments can be proctored with the presence of professionals familiar with the instruments assessment, but also delivered in a self-paced and self-administered mode in which participants follow instructions supplied directly by the assessments. The lack of human proctors calls for appraisal for test validity due to higher chance in deviation from experimental protocols (Shields et al., 2021).

There has been a wealth of research into the impact and equivalence between computerized and paper-and-pencil testing (Clinton, 2019; Delgado et al., 2018; Mead and Drasgow, 1993; Neuman and Baydoun, 1998; Wang et al., 2008). However, the development, deployment, and validation of online assessments for children come with their own set of complexities. In this paper, we focus on the logistical and technical considerations in building an online assessment platform in a child bilingual and cognitive assessment context. As computerized tests go online and are remotely delivered, additional challenges arise (Ramsey et al., 2016), including lack of supervision, attention, connection, diversity in testing environments, and maintenance. During the online conversion of paper-and-pencil tests, researchers are also concerned about how much of the data collection process should or can be automated, what extra features are included, and what pitfalls can ensue during the development and data collection. We discuss these issues through our experience developing the *Arithmetic, Chinese and English for Kids platform* (the ACE Kids platform). In this article, we focus on building upon the current understanding from existing work on digitizing paper-and-pencil assessment, by highlighting important fundamental considerations, but also offers new insights toward concurrent assessment of bilingual literacy markers that have been integrated in the same interface and database workflow. In addition, we discuss the practical considerations of collecting a large matrix of assessments (40 + in the case of ACE Kids) alongside digital metadata to promote the collection of usable, valid and dimensionally rich datasets.

## 2 When to build a custom platform

With the ubiquity of digital devices and internet connectivity, online assessments offer unparalleled convenience and flexibility for both educators and students. Several online assessment platforms have emerged, each with its own unique features and methodologies. These include school-facing brief, targeted screening tools such as DIBELS (University of Oregon, 2020) and MAP Reading Fluency (Peng et al., 2024); Research-grade, diagnostic and profiling tools (with digital administration options) such as Woodcock-Johnson IV (Schrank and Wendling, 2018) and WRAT-5 (Wilkinson and Robertson, 2017); as well as rapid (usually 3–15 min), self-administered online assessment such as ROAR (Yeatman et al., 2021), RISE (O'reilly et al., 2012; Sabatini et al., 2015), WordSword (Ho et al., 2024). These options were variously designed for large-scale data collection to screen, diagnose or establish psychometric properties of participants. An online assessment platform is particularly versatile for large-scale, longitudinal studies assessing the trajectories of reading and cognitive abilities in children. Experts in learning management systems have supported the feasibility of reading assessment and the organization of assessment platforms (Algiovan, 2022). For participants, these platforms allow easy and flexible access to the assessment materials.

Literacy assessments often encompass a variety of literacy skills, such as orthographic, phonologic, and semantic knowledge, as well as executive function skills such as working memory and attentional control (Kim, 2020). Therefore, a comprehensive literacy assessment can be lengthy. Typically, a language and literacy evaluation takes about 3 to 4 h for a younger child (Dyslexia Help, 2011). In contrast, one estimate is that young children have a typical attention span of 5 to 7 min for those aged 6 to 7, potentially impacting the validity of lengthy assessments (Asprilia et al., 2020). Assessment platforms allow children to finish the individual tests in different sessions, which can mitigate the effects of mental fatigue and limited attentional span. Another prominent challenge of assessing young children’s reading abilities is adapting reading passages and questions to suit various age groups and reading levels, as content must be engaging and relevant to all children. Building a customized assessment platform for reading can enhance the interactive experience and engagement for children of different ages. Incorporating interactive elements, such as multimedia content, games, and adaptive learning algorithms, can enhance the perceived quality of experience toward reading assessment and lead to optimal performance and increased validity (Kougioumtzidis et al., 2022; Navarro and Mourgues-Codern, 2018).

Given these advantages of online assessment for reading and mathematics, we focus on how best to develop an assessment platform for school-aged children. In order to collect a large amount of behavioral data from children, we identify three approaches: traditional in-person data collection, serviced online tools, and customized assessment platforms.

### 2.1 Traditional in-person data collection

Traditionally, data collection has been conducted at home, school or lab settings. Experimenters administer tests using printed test materials while recording and rating responses. The approach is familiar to researchers, and yields reliable and valid data. Close monitoring is also possible. However, professionally-trained proctors are often necessary to ensure adherence and comprehension of materials. It is also logistically challenging to arrange for the collection of a large and diverse sample (Li et al., 2024), reducing scalability. Human errors are also common due to the need to manually collect, score and process responses (Acharya K et al., 2022; Miller et al., 2009).

Other than data collection on-site, participants can also be tested in a laboratory environment. It allows researchers to leverage static equipment. This reduces costs, and allows for calibrated measurements. The latter facilitates more complex designs and capture of additional variables. It also reduces experimenter commute time and expenses. However, space then becomes a limiting factor as the data collection scales up. The need for participants to travel might also discourage participants from signing up.

### 2.2 Serviced online tools

Researchers may opt to transition to an online mode that leverages experiment builders, many of which are fully serviced for psychometric needs (e.g., Gorilla, Inquisit, Pavlovia, or MIT Lookit (Scott et al., 2017; Scott and Schulz, 2017); for a review, see Sauter et al. (2020)). Online solutions reduce costs by reducing the need for experimenters, training and venues. This reduces project cost and allows for a larger sample size (Hartshorne et al., 2019; Li et al., 2024). It is more scalable than non-web-based solutions (Sonwalkar, 2013), which means capacity can be adjusted flexibly according to sample size. Online solutions also potentially allow data collection from a more diverse sample. Digital delivery enables a high degree of standardization in presentation (Sochat et al., 2016). Automated scoring of online responses also reduces human errors (Acharya K et al., 2022; Yarnell and Pfeiffer, 2015). Last, performance anxiety might lessen in assessments delivered in a computerized manner (Abanobi et al., 2024) compared to paper-and-pencil tests.

### 2.3 Customized platform building

Researchers can last opt to develop a customized assessment platform. One important motivation is that such a platform has a high flexibility. It is easier to implement custom functions, such as direction of participants between assessments; report generation; or response capture in multiple modalities; Gamification elements can also be added to improve engagement and boost intrinsic motivation (Lumsden et al., 2016), though care should be exercised with direct integration as gamification might lead to anxiety (e.g., if leaderboards are included) (Zhang and Crawford, 2023), and inappropriate test-taking strategies unrelated to the assessed skills and construct-irrelevant variance (Friehs et al., 2026; Reed et al., 2020). External modules, like custom speech analyses, can be easily connected to a customized platform. Researchers might also capture metadata to understand the usage situation and tackle shortfalls in monitoring.

Another important motivation for developing a customized platform is increased control. Researchers can be assured of full control over software, servers, data and security. Sometimes, project proposals also call for the creation of a standalone product without rights issues, and to minimize reliance on existing tools in order to establish a clear impact and unique contribution. Reliance on third-party hosting and tools might also mean reduced control over data. Behavioral assessments involving sensitive personal data usually have stricter data ethics and privacy concerns (Favaretto et al., 2020), which could complicate data handling on non-local locations.

While a customized platform promises higher flexibility and control, software development of online, computerized assessments require significantly more initial time and monetary investment compared to developing traditional, paper-based assessments (Luecht, 2006; Triantafillou et al., 2008). This offsets time and cost advantage from labor cost and administration overhead. There is also a need to conduct validation (Gil et al., 2015; Neumann and Neumann, 2019; Wang et al., 2008) in order to ensure validity and reliability of online delivery. This validation can potentially be lengthy and resource-intensive. The need for a diverse expertise in programming and design also challenges research teams. Meanwhile, outsourcing likely increases the turnaround time significantly, and increases communicative burden. In either case, costs associated with ongoing monitoring and maintenance are ever-present. A further comparison between the advantages and limitations of traditional vs. online assessment is presented in Table 1.

**Table 1:** Comparison of advantages and limitations of online vs. traditional testing in child assessments.

Concerns in child assessments	Online testing	Traditional testing	References
Speed of result delivery	Faster	Slower	Cottin et al. (2022); Heiervang and Goodman (2011)
Degree of reach	Anywhere with online connection	More inflexible, either on-premise in research labs, schools or homes	Gustafsson et al. (2021); Tandon et al. (2026)
Engaging features	More diverse (sounds, colors, animations, gamification elements)	Less diverse	Pramana et al. (2018)
Level of Distraction	Potentially more	Usually lower	Shields et al. (2021)
Comprehension due to delivery method	Potentially challenged by screen inferiority effect and working memory strain due to scrolling	Less susceptible	Clinton (2019); Salmerón et al. (2021)
Tech issues	Internet drops, application glitches, poor typing, inexperience with device	Less affected (especially if delivered by trained personnel)	Tabachnick et al. (2022)

These considerations are discussed below and punctuated by our experience in developing our own assessment platform – the ACE Kids platform, which began in January 2022 and entered the phase of formal data collection in April 2024. The ACE Kids platform is designed to collect behavioral data from 1,500 Hong Kong Chinese children from kindergarten year 3 to Grade 6, as well as their parents, covering domains including Chinese and English language skills and mathematical skills. Assessing children as young as K3 aids the early detection of linguistics and cognitive markers associated with learning difficulties (such as dyslexia), and the seven-grade time window from K3 to Grade 6 gives a thorough coverage of the critical developmental period of Chinese, English and mathematical abilities. The children were tested yearly, meaning that a longitudinal tracking of the trajectories of developmental markers is possible. Over 800 participants completed at least one wave (full completion of all measures during a time point) as of August 2026. The assessments included were adapted from previous projects probing the behavioral, genetics and neuro-markers in Chinese, English and mathematic achievement in Hong Kong Chinese children (e.g., Pan et al., 2023; Ruan et al., 2023). The assessment platform includes over 40 measures (see Appendix A) made for children, with a subset adapted for parents. There are also questionnaires probing demographics, family and metacognitive factors.

The ACE Kids platform provides additional insights as it consists of analogous tasks focusing on logographic (Chinese) and alphabetic (English) processing strategies, allowing a deeper investigation into overlaps and unique contribution of biliteracy during early childhood development. Therefore, we also highlight how to effectively conduct concurrent online assessment of analogous literacy and cognitive markers in the same interface and database workflow, such as unifying the interface grouping and presentation of assessments focusing on analogous bilingual markers. We also highlight how digital testing both necessitates and allows a more nuanced understanding of testing environments, such as different device modalities, e.g., desktop vs. laptop vs. tablets; handwriting tasks using fingers vs. stylus; The ACE Kids platform collects such usage metadata that allowed for subnorming to be conducted. The large matrix of 40 + tasks allows for a more comprehensive fingerprinting for cognitive and literacy development that go beyond the traditional investigation in longitudinal and transfer effects, but also presents challenge in attention retention and effective data collection, and so we highlight recommendations when building and administering a large battery with multi-hour assessment, in a digital, online, self-directed and children-oriented context.

## 3 How to plan for technically informed assessments

Given the significant costs and time involved in developing a customized assessment platform, it is important to commit only when it is necessary and brings more benefits compared to costs. The following section discusses the fundamental considerations at planning and proposal drafting stage. This includes (1) project goals and scope, (2) programming environment, and (3) hosting and maintenance.

### 3.1 Project goals and scope

Project goals and scope need to be clarified at the outset of the project. Specifically, the number and types of assessments are important. As the total length and number of assessments increase, they contribute to participant exhaustion (Ackerman and Kanfer, 2009), thus it becomes less practical to collect data over a few sessions. Online assessments in contrast allow participants to be tested over multiple sessions in a self-paced manner. The types of assessments also matter. A richer range of modalities can be captured through increased customization. Examples include collection of both typewritten and handwritten responses, which involve different perceptual and cognitive processes (Longcamp et al., 2008; Longcamp et al., 2005); or speech responses which can then be further transcribed and analyzed automatically. In comparison, for assessments that consist of tasks with simple designs in singular modality or plain questionnaires, serviced online tools (e.g., Qualtrics) or offline solutions (e.g., E-Prime or PsychToolbox) can be more cost-effective.

Researchers should also consider the time needed to develop and validate tasks, which scales up with the number of assessments, modalities, and features. Development can occupy a significant portion of the project life cycle, which means there will be less time for data collection and analysis. Contrasting the development of paper-based assessments, online tasks require additional testing and updates, often arising from needing more effective instruction, stimuli and feedback in an unsupervised environment. If the target population is young children who tend to have a shorter attention span (Simon et al., 2023), further engagement-retention features like gamification (Khaleghi et al., 2021) might be needed. Last, researchers should consider whether an online platform calls for work beyond project duration, such as continued maintenance or feature development, which can benefit future projects but also increase resource commitment.

### 3.2 Programming environment

To deliver customized online assessments, the assessments have to be programmed, and then hosted on the internet and accessed by participants. This can be done through the creation of applications, such as web applications and mobile applications. Web applications are programs that are stored on remote servers, delivered online and then rendered to users through their browsers (Taivalsaari et al., 2008). Mobile applications are programs that are written to run on mobile devices such as tablets and smartphones (Kortum and Sorber, 2015).

For a team looking to create a customized assessment platform for the first time, developing a web application can be advantageous due to cross-platform support and a gentler learning curve. First, web applications work well across different system environments, since they are rendered through browsers that are cross-platform. The web application of the ACE Kids platform is a Python application written in Flask (Grinberg, 2018). Flask is a lightweight web development framework that has a simple structure (Singh and Akash, 2022). This makes it suitable for researchers to quickly produce a working platform. For researchers looking for further ease of use, JATOS (Lange et al., 2015) and Pushkin (Hartshorne et al., 2019) are tailored tools that come with more experiment deployment, user and data management functionalities.

Second, there are JavaScript libraries such as jsPsych (De Leeuw, 2015) and PsychoJS (Peirce et al., 2019) that are built for browser-based applications and help with precise psychometric assessments. Meanwhile, building native mobile applications calls for an advanced level of programming expertise. Novice mobile application programmers might take up to 3 years to be deemed experienced (Dobrean and Dioşan, 2021). In contrast, browser-based libraries for psychometric tests are utilizable even by programming novices (Lukács and Haasnoot, 2023). The ACE Kids platform utilizes the jsPsych 6.3 library (De Leeuw and Gilbert, 2020). The jsPsych library provides accurate response time on par with offline experiments (Pinet et al., 2017). The use of open-source libraries such as jsPsych also allows modification for specific use cases. Such modifications include packages developed by the MIT Lookit team to be used with jsPsych (Green and Gilbert, 2024), or the integration of handwriting and browser speech recognition results into the jsPsych data object in ACE Kids. A more detailed description of the platform system environment is included in Appendix B.

Beyond programming expertise, design expertise is also important for presentation and styling, including arts assets creation, such as animations which convey engaging instructions. On the ACE Kids platform, art design was conducted entirely in-house, except for the production of parental instruction videos that were outsourced to provide professional voiceover and animations.

### 3.3 Hosting and maintenance

Server hosting has important implications for budgeting and scalability. Hosting can be done via self-hosting or third-party cloud services. Self-hosting involves procuring physical hardware to host the application online. It allows the highest degree of freedom in programming environment. It also ensures sensitive data are kept only within the team. However, self-hosting requires a broader system administration expertise (Gröber et al., 2023; Molnar and Schechter, 2010). Self-hosting also means full responsibilities over hardware, maintenance and security (Molnar and Schechter, 2010). Scalability is constrained by hardware infrastructure. These factors make self-hosting potentially cost-inefficient. A variation to self-hosting is to host the web application on the network of an attached institution. It retains similar advantages while offloading custodial responsibilities. Institutional infrastructure can provide faster, more secure and cheaper hosting for attached researchers (Almajalid, 2017). The Flask application for the ACE Kids platform is hosted on the departmental server running Ubuntu 18.04.5 LTS in the psychology department of the attached institution, which incurs no active maintenance or subscription costs to the research team.

Researchers can also opt for commercial cloud services to host their web applications, such as Amazon Web Services (AWS), Microsoft Azure and Google Cloud Platform (GCP). This provides scalability as capacity can be expanded on demand. This also means costs are metered against usage (Rappa, 2004). Accurate estimation of costs at the proposal stage of a project can be challenging, since a broad technical expertise is required, and prototyping might only be possible after project start. This also imposes a need to maximally optimize the web application, since even small inefficiencies and misconfiguration might increase costs significantly (Henderson, 2006; Yang et al., 2019).

A web application hosted on a server requires continued maintenance to ensure reachability and usability. Server administration involves overseeing the network activities and data security on the server (Scarfone et al., 2008). Unless full self-hosting is used, this level of oversight can be delegated. However, the work of programming does not end at launch, and continues over the project lifespan, due to the potential need to fix, update and expand the platform.

The metadata – granular details of participant activities on the platform – also requires monitoring. This can be tracked via server access logs (Suneetha and Krishnamoorthi, 2009), but graphical interfaces or message alerts are more accessible for general researchers. For example, JATOS offers study and application logs for activities (Lange, 2024). For the ACE Kids platform, participant access, trial completion, and task completion are viewable on the administrative interface. Important milestones such as the start and end of assessment, user reports and idleness also trigger automated email reports to the research team.

## 4 How to design child-facing assessments

The interface design of a customized assessment platform, or the frontend, refers to the visible parts participants see and interact with. The frontend directs participants to complete the assessments, and yield data to be sent to the backend where the web application and database reside (Figure 1). To illustrate the concrete implementation and child-facing considerations via the case in ACE Kids, the basic parts of such a frontend is explained below.

**Figure 1:**
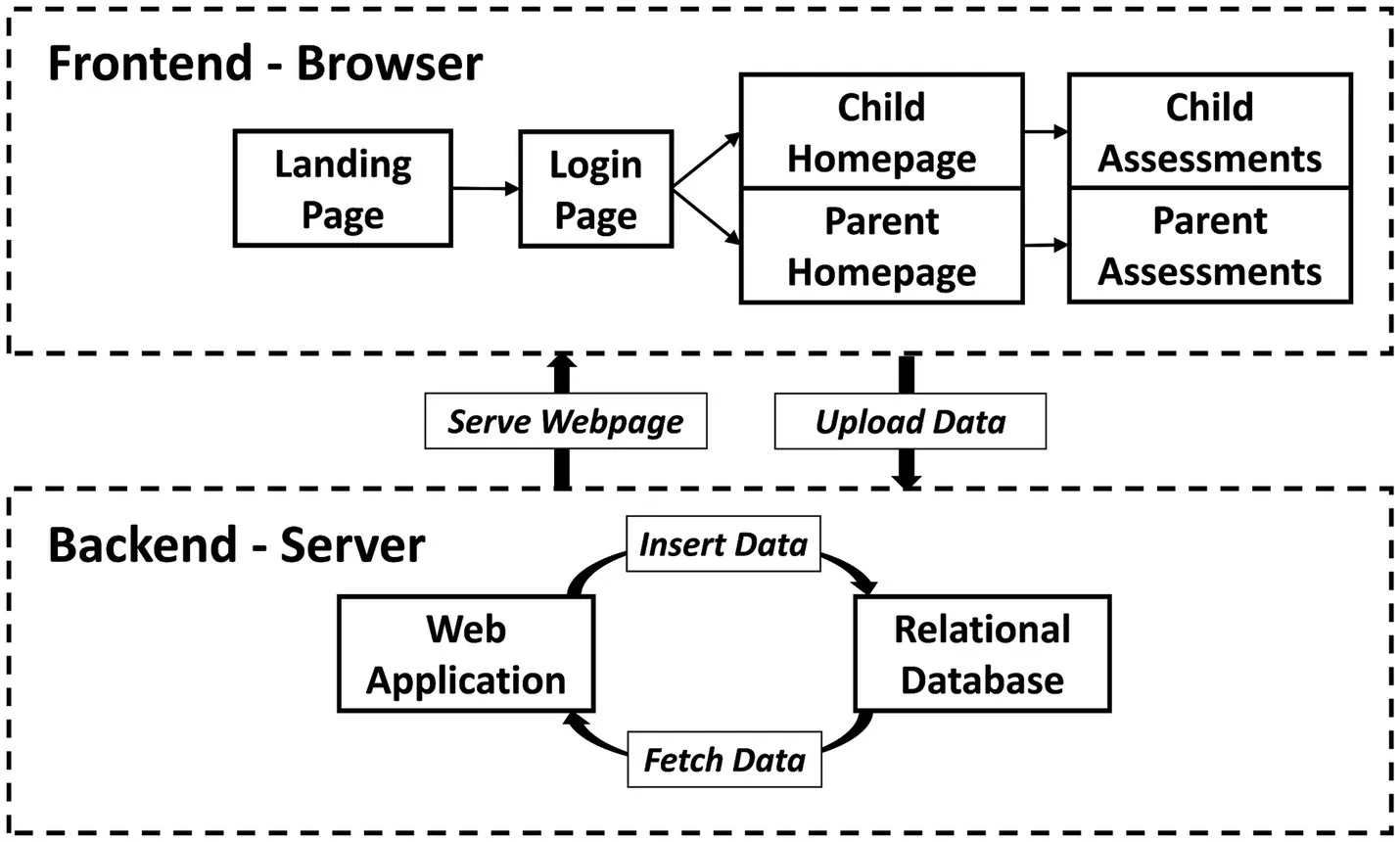
Programmatic representation of the ACE Kids assessment platform. The platform consists of the frontend, which is the participant-facing webpages rendered in browsers, and the backend, which is the server where the web application and the relational database reside. The web application controls which page is shown to participants, and delivers user-specific data to the frontend. The frontend collects data from the assessments and uploads the data to the server. The data is organized by the web application and inserted into the relational database according to each user, task and attempt, alongside other metadata.

### 4.1 Interface design

The landing page (Figure 2a) is the first area visitors see. It introduces to them the platform and directs participants to assessments. On the ACE Kids platform, child users are directed to the login page through large visible buttons. There are also instructions for first-time visitors for the sign-up process. Social media integration is also present, where project news is disseminated. The background, team and goals of the project are also available.

**Figure 2:**
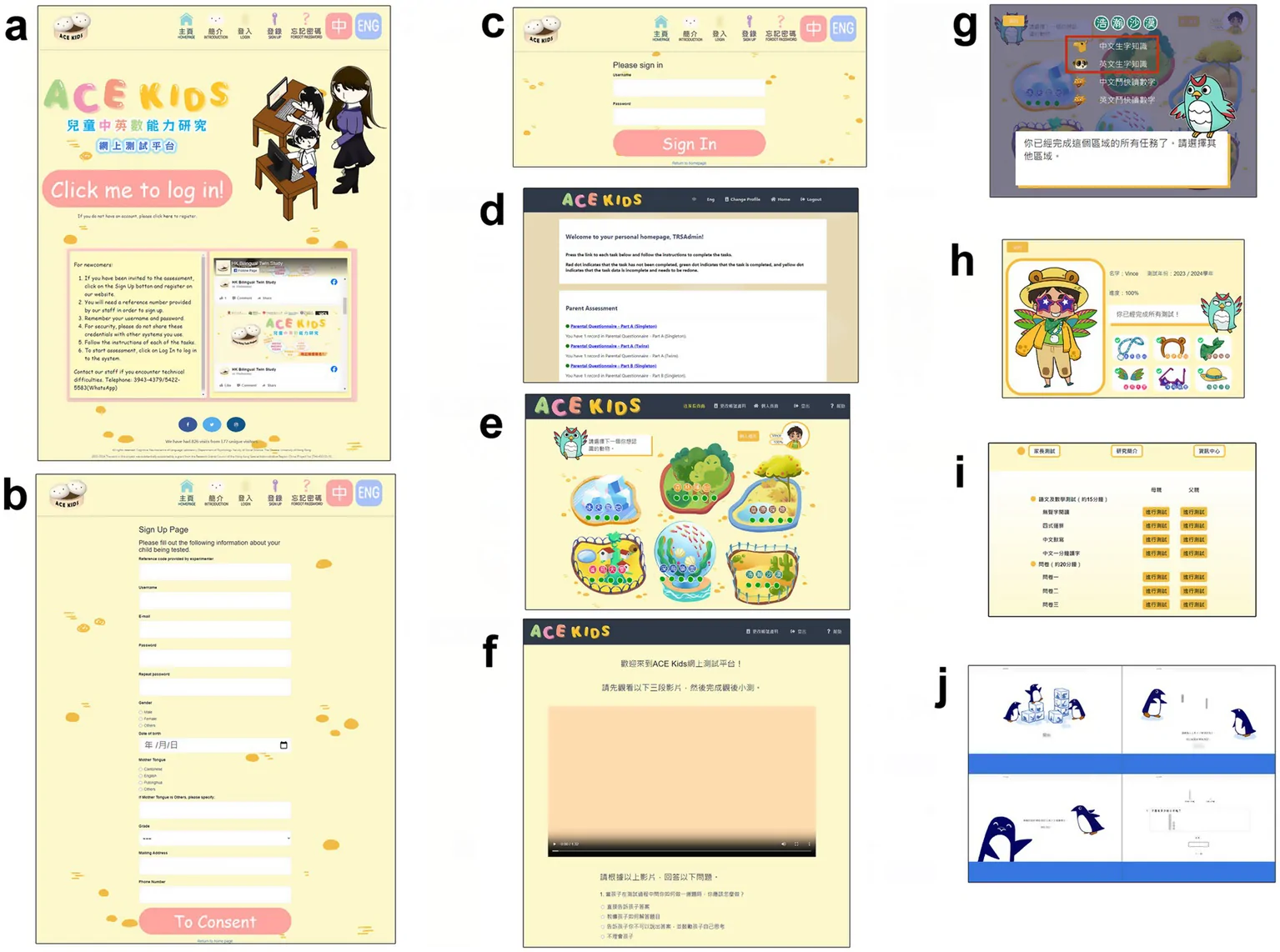
Screenshots of the ACE Kids assessment platform. **(a)** Landing page with usage instructions and social media integration; **(b)** Sign-up page; **(c)** Login page; **(d)** Partial screenshot of legacy personal homepage; **(e)** Gamified child personal homepage, with six themed areas; **(f)** Partial screenshot of parent tutorial page with videos and quiz; **(g)** Desert-themed area with Chinese and English Vocabulary Knowledge tasks (highlighted in red rectangle) bundled together; **(h)** Avatar and trophy page; **(i)** Parent assessment page; **(j)** Example of in-task interface, with cartoon artworks and themed decoration.

The sign-up and login pages (Figures 2b,c) are the front-facing part of user access control system. On ACE Kids, participants sign up through the sign-up page, with a unique reference code given by experimenters. After registering a user account, participants may login with their credentials. User access control allows attribution of assessment data to unique participants, and allows customization of task presentation and progress tracking.

The user homepage is where participants access different assessments and observe overall progress. In the ACE Kids platform, we had initially developed a simple user homepage (Figure 2d) allowing access to all assessments at a glance. However, this was superseded by a newer gamified interface (Figure 2e) after further user experience studies during the alpha testing stage (detailed in the next subsection). The new homepage consists of three parts: parent tutorial, child homepage and parent homepage.

Parent tutorial (Figure 2f) is an introductory module to familiarize parents with their expected role and conduct during child assessment. It consists of three instruction videos and a short quiz. Parents are instructed to attend to challenges that arise when children use the platform. They are told to refrain from directly intervening unless the issue is related to technical difficulties. They are also instructed about basic troubleshooting, such as managing microphone permission on the browser for assessments with a speech component. The videos and the short quiz serve to communicate the basic expectations for parental roles, which align researcher goals for valid data and parent goal for accurate child report (Appendix C) generated from said data.

In the gamified child homepage, children are introduced to a cartoon owl who guides the users through assessments. The individual assessments take anywhere from 3–15 min, and one ‘session’ – a single sit-down period of which a child autonomously complete a series of assessments on their own initiative – can last anywhere from the duration of one single task, to however long a child can persist in one single sitting, usually anywhere from thirty minutes to a few hours. To account for the weaker abilities for younger children to remain alert and attentive over longer periods of time, we have reminders for children to take a break, which show up on the home screen every thirty minutes. Generally, a child is expected to finish the entire battery of assessments in an accumulative amount of 4–5 h, paced in as many sessions that they need within four weeks.

Assessments are grouped together into areas by their similarity and time needed. For instance, the Chinese-version and English-version of the Vocabulary Knowledge tasks are grouped together in the desert-themed exhibit (Figure 2g). This visual grouping facilitates biliteracy assessment of analogous structures and rules, and reduce the time involved in familiarizing oneself with the interface and task structure as presented on the platform when the children complete the tasks sequentially.

Participants are also able to collect trophies which can be equipped on customized avatars (Figure 2h) as extra motivation to complete all tasks in an area. The goal is to increase engagement and persistence of children by providing a gamified outlook (Mohammed et al., 2017). The parent homepage (Figure 2i) presents a list of assessments to be completed by the mother and/or the father. It also allows the parents to re-watch the parent tutorial videos, to revise upon the duties, expectations and rules of conduct during assessments.

### 4.2 Assessments

Assessments are the heart of the platform. They collect participant data; and an effective data collection relies on well-designed assessments that minimize the technical and physical hurdles for participants. This is done through clear instruction, intuitive navigation and frustration-reduction techniques, which is illustrated below with ACE Kids as an example.

In ACE Kids, the presentation of tasks is standardized using the jsPsych library. The tasks generally consist of three parts: instructions, practice trials and experimental trials. We include guidance to minimize any confusion. For instance, we included a progress bar in most tasks. We use large, clear fonts, buttons and images wherever possible. Instructions are voiced, accompanied by animations or videos. Practice trials familiarize participants with the task interface and demand. Participants receive feedback about their correctness during practice, and consecutive incorrect answers yield a prompt showing the correct answer. Interactions are mainly done via button clicking. This minimizes the need to switch input modes frequently and makes the platform more touchscreen-friendly. The beginning of practice, experimental trials, response recording, and the end of a task are all clearly shown. As part of gamification, art assets such as cartoon characters and backgrounds are also added to the task screen (Figure 2j). This brings the visual style of assessments in line with the gamified homepage, while maintaining continuity with non-gamified paper-and-pencil assessments.

To ensure a smooth usage experience and data quality, small-scale pilot tests can help identify bugs, chokepoints and helpful features. For ACE Kids, we ran multiple rounds of internal tests and pilot tests. The pilot tests were different to the actual assessments in length, coverage and format, and focused on user experience and included quantitative measures to probe the subjective ease of use during platform use.

To illustrate the utility of these tests, we highlight one such pilot test involving a partial battery of tasks in October 2023, where we recruited 24 pilot participants from kindergarten to Grade 6 (Mean age = 8.83, SD = 1.95). They each autonomously completed two to five tasks out of all child assessments included in Appendix A, each taking approximately an hour in total. Each task included in the large battery was tested at least once within the sample of pilot participants (who each complete a portion of the entire battery), each task is tested up to three times within the sample. This led to the pool of 24 participants completing 99 incidences of the 45 included tasks (as of 2023). This approach was logistically light but was limited methodologically because every individual participant received different sets of tasks, thus the scores in the questionnaires derived represented less of individual task characteristics, and more of the general jsPsych-based computerized implementation of the paper-and-pencil tasks that was uniform across the tasks. The experimenters did not intervene unless the children were seen struggling to proceed. Experimenters noted the following: (1) estimated length of attention span; (2) frequency and causes of distractions; (3) whether or not participants could understand the instruction and complete the practice trials by themselves; (4) environmental interferences during the test.

A questionnaire (Appendix D) was also completed by the children. The questionnaire consisted of three parts, the first part being the Cognitive Load scale (Paas, 1992) which probed the mental effort spent in completing each assessment on a 1–9 scale (e.g., ‘In the game I just finished, I invested very, very low mental effort (1) – very, very high mental effort (9)’); the second part is the Extraneous Cognitive Load scale (Leppink et al., 2014), in which participants were asked about how easy or difficult it was to interact with the assessments via the device and platform (e.g., ‘Using the electronic monitor to read is very, very easy (1) – very, very difficult (9)’ or ‘The audio instructions during the test were very unclear’); the third part records the usage environment, including the type of device and operating system (e.g., desktop with mouse, laptop with touchpad, tablet), and the device the participant most commonly used in their daily life.

We found that participants averaged at 4.81 (*SD across tasks* = 2.46) for the Cognitive Load questionnaire and at 3.42 (*SD across participants* = 1.12) for the Extraneous Cognitive Load questionnaire. Of note is that the statistics of Cognitive Load questionnaire was derived from the 99 item scores, since it captures across-task variabilities better than computations done on participant scores; whereas statistics of Extraneous Cognitive Load was computed from scores averaged across items of each participant, as the questionnaire items already represented general user experience toward the tasks. Regardless, the scores in general only reflected an intermediate indicator of the pre-optimized and pre-gamified state of the assessments and platforms; instead of taken to be a full usability assessment, it served mainly to give a general qualitative overview of the tasks’ usability at that stage from participant point of view. For example, we relied on the elaboration question in Part 2 of the questionnaire in Appendix D to glean feedback to inform subsequent assessment design and gamification. This was our immediate concern during that time (September 2023) of the platform development, and included here as a demonstration of an example of incremental improvement measure that can be implemented in a project cycle.

As such, we referred to participant feedback and identified tasks which participants indicated to require high mental effort and streamlined the instructions, for example, by breaking lengthy instructions down into multiple pages, rephrasing instructions, or adding multimedia instructions. We also collected responses from children regarding factors affecting their extraneous cognitive load, such as font size and interface elements, and made improvements. We collected data on device usage and found that 20 out of 24 children most often used tablets or phones daily. The discrepancy between their familiar digital environment and testing environment was also considered, so we aimed at improving responsive web design (Baturay and Birtane, 2013) to make the platform more mobile-friendly and resemble their ordinary usage environment.

From the experience with this evaluation during the alpha testing period, we therefore implemented design choices to especially benefit younger children (K3 to grade 3), who might not be as versatile with input methods or used to reading long instructional texts. We adopted a shift from keyboard-based input (e.g., pressing a key to continue, as lab-based computerized assessments often do), to a button-based or touchscreen-friendly input. We enlarged and color-coded important interface elements such as the next and record audio buttons. We also added more audio readouts to instructions and text-dense questionnaire items. In tasks involving audio recording, which can be more vulnerable to interferences in home environment or device issues, we implemented replay and redo mechanisms for fault tolerance. And through the aforementioned parent tutorial system was also developed with the experiences gained through the evaluation.

We further implemented frustration-reduction techniques, features aimed at making the experience of the long assessment more viable for sustained attention. This includes inclusion of practice trials which are low-stake practice loops before the actual trials to allow mastery of the requirements of the assessment and the interface. We added skip options for frustrating items (e.g., at half point of longer tasks, e.g., Chinese Character Reading with a total of 160 items, balancing between frustration reduction and non-inducement of early skipping) as a basic adaptive approach. We followed the trial-based single-question layout of jsPsych to minimize distraction, and our assessment layouts (bottom right for Figure 2j) are designed to be relatively uncluttered (compared to the gamified homepage) to promote focus.

## 5 How to manage data quality and security

An uninterrupted data flow from the user to the database is critical for successful data recording. This relies on successful integration between the user, the frontend and the backend. The frontend renders the effective instructions and assessments on webpages and collects behavioral data from the user. It handles direct interactions so assessments can be delivered across different users and devices. This data is then sent to the backend. The web application serves as a nexus in which data collected from the participants are organized according to user and task, and sent to the database. The web application centralizes data processing and manages user authentication for privacy, so that data are sorted and labeled correctly. The database stores the data of the user and assessments, and dispenses the data when called upon by the frontend. The database for the ACE Kids platform is a relational database that is run on the MariaDB 10.3.28 (MariaDB Foundation, 2021). A relational database allows for data to be structured in an interrelated manner while providing security from data encryption (Shmueli et al., 2014). Within data flow management, we highlight two specific aspects that require attention, namely response scoring and data integrity.

### 5.1 Response scoring

Response scoring refers to how the frontend and backend work together to record and code the behavioral responses made by participants. Online assessments allow responses to be graded automatically, but the ease of implementation is not equal across cases. For instance, multiple-choice, cloze-type or matching items have a fixed number of possible answers, therefore automation of scoring is trivial. But when it comes to constructed-response items, or items requiring speech responses or handwritten responses, automated scoring becomes more challenging. In ACE Kids, the behavioral data collected typically include the Reaction Time (RT), the raw input (multiple choice answer, typed answer, handwriting image and stroke order, recorded audio speech), and the automatic grading wherever it is applicable (See Appendix A for individual task descriptions; Appendix B for database implementation).

Researchers may devise a scoring scheme for constructed-response items, in which certain keywords or patterns are scored mechanically. However, for more contextual grading criteria, manual scoring, or leveraging of large language models to automate grading (Lee et al., 2024) might be more suitable. Analogously, speech responses can be transcribed via browser-supported recognition functions, which reduces programming and hosting overhead. The recognition results can be compared to model answers in real-time. However, each browser uses their own speech recognition engine (Ross, 2017), which is generally close-sourced and not fully configurable by researchers. Alternatively, researchers may aim at training their own speech models (e.g., Li et al., 2019; Li et al., 2020) that provide a higher methodological transparency. It also provides flexibility toward different task demands, such as recognition of non-real phonemes. This, however, requires an understanding of techniques in machine learning, and how to apply them in audio classification and recognition (van Niedek et al., 2016). In both cases, real-time grading and recognition through machine learning also require additional expertise and computing resources.

### 5.2 Data integrity

Data integrity refers to the steps taken to ensure the data is recorded correctly and completely. It also covers data access as well as security. On the frontend, researchers should ensure the assessments are presented as intended across all environments, and that data is captured and sent to the backend correctly. Then on the backend, the data must be correctly parsed and inserted into the database. Since this process is normally opaque to the participants, it is important to provide feedback to participants as well as experimenters about whether participants have successfully engaged with an assessment and generated valid data.

On ACE Kids, data are sent from the frontend and logged in the database on completion of each trial, instead of logged in bulk at the end of an assessment. This allows retaining of as much data as possible in case of hardware or software failure. Premature attempts to quit an assessment yield a warning message that the data recorded will be incomplete. Participants are also shown the completion progress of each assessment. They can notice if there are discrepancies between their efforts and records. Experimenters also receive email reports about participant progress, including partial and complete attempts. Participants have four weeks to complete all assessments on the platform, and an alert is sent to experimenters if participants exceeded this time without full completion.

For assessments involving speech input, we implemented measures to ensure the recording of intact audio. Detailed instructions are given during parent tutorial and assessments regarding giving browser permission for audio recording. During the trials, participants are cued by auditory beeps indicating that they might start speaking. Failure to engage with the speech recording function triggers a warning screen. The raw audio data of each trial is also recorded and stored alongside automated recognition results.

To examine and ensure data completion, experimenters can easily, through the web application of ACE Kids, fetch data from individual assessments from the database, package the data across multiple assessments into a summary, or to back up the entire database for restoration.

## 6 How to validate online assessments

Validation refers to the steps taken to establish the equivalence of online assessments with paper-and-pencil predecessors, and ensuring the former is valid and reliable. This includes examining the online assessment’s convergent validity and criterion validity (e.g., Lam et al., 2013) so that the assessment correlates with related measures, as well as predictor and outcome variables. Establishment of equivalence often involves collecting data from parallel measures from the same population, and then comparing their psychometric properties (Ho et al., 2024). For example, a high intra-measure reliability and inter-measure correlation (Joubert and Kriek, 2009) point to a reliable and valid assessment. For online assessments, validation is an important step to establish continuity with previously collected behavioral data. Here we highlight four concerns in the validation process: Logistical transition, assessments adjustment, contextualized norm and usage rights.

While the data collection phase of the ACE Kids project is still ongoing and yet to conclude as of the time of writing (August 2026), we have collected preliminary evidence on validity of component assessments using the data we have collected thus far. This included Shin et al. (2026) which stated that cognitive-linguistic skills captured by the online measures in the platform were able to account for 37% of variance in word reading performance for grade 1–3 and 47% for grade 4–6. Ho et al. (2024) also compared the Silent Word Reading / ‘WordSword’ task, comparing between the paper-and-pencil version and online version and found correlations above 0.8 between the versions. Whereas the validation results of the wider battery and a more comprehensive validation with formal methodology are pending, we aim to proceed to mostly focus in this paper the principles of assessment platform design, and data collection planning that would facilitate such validation attempts.

### 6.1 Logistical transition

Conducting validation of online assessments often necessitates collection of paper-and-pencil data in parallel. Researchers need to factor the additional time needed and resources for both modes of data collection. If parallel forms are issued to the same set of participants to establish correlations, the separation in time between the two modes of assessment, and the practice effects introduced by multiple testing must be considered. Participants might experience carryover effects and practice effects (Greenwald, 1976) if within-subject validation is used. Alternatively, researchers may adopt a between-groups approach by issuing the tests to separate subsamples and comparing the psychometric properties in both tests.

In the case of ACE Kids, yearly assessments are conducted for participating children, and a subsample of children are selected to complete at four weeks after the conclusion of the last completed task, during which onsite personnel would also conduct saliva collection (for genetic analyses) and also IQ testing. The composition of tests administered is generally consistent across grade levels, except K3 children who complete a special easier version of the arithmetic task in the form of numeric comparison and addition task (see Appendix A), as well as skipping the more word-dense Mathematical Word Problem task and Chinese Comprehension Task. Meanwhile, another subsample of children conducts all paper-and-pencil assessments and they then transition into online assessment in the next year. We keep with a faithful online replication of the paper-and-pencil version, instead of adopting a more flexible and adaptive presentation, mainly in consideration of the parallel-form equivalence between the two version; we kept the number, content and testing order of tested items equivalent, so that we can first establish the utility of the same materials in an online environment, before further adaptivity is considered and added. These sets of data therefore allow for examination of correlations and temporal stability of parallel scales, balancing between the consideration of carryover effect, practice effect and examination of cross-format equivalence.

### 6.2 Assessment adjustment

Adjustment of assessments can arise due to unique demands of online environment. An online presentation means automated feedback and no direct human supervision. There are also diverse usage environments and possible technical constraints or errors. They necessitate measures to prevent or ameliorate the issues. For example, in paper-and-pencil assessments, stopping rules are often applied to terminate an assessment after a specific number of consecutive errors (e.g., Cheung et al., 2020; Tong et al., 2017) to reduce testing time and burden on children. However, this depends on whether the platform can reliably judge correctness in real-time, otherwise stopping rules will not be applied consistently, leading to premature termination. On ACE Kids, we removed stopping rule criteria for speech tasks and opted to present all items; and in cases we identified that time constraint and participant fatigue became an issue, we also trimmed the number of total items after item response analyses; This is because browser speech recognition, while lightweight, might not always render correct real-time results, and retaining all trials and recorded audio allow for accurate postprocessing. Online test administration imposes a lighter time constraint, since participants can pace themselves across assessments, therefore presentation of more trials is plausible.

### 6.3 Contextualized norms

Establishing norms for online assessments enables interpretation and contextualization of scores obtained from assessments (Lenhard et al., 2018). If validation reveals equivalence between online and traditional measures, then an argument can be made for referencing the traditional norms when interpreting a score. However, if the validation process reveals significant differences in psychometric properties, then there is a need to establish a separate online norm. Conversely, deviations in norms can also inform researchers about the difference in parallel measures. If online norms are significantly lower than paper-and-pencil norms, it might imply there is increased cognitive load (Noyes and Garland, 2008) possibly due to instructional or technical challenges associated with online administration. This calls for clarifying instructions, optimizing interface, and providing support. But if the online norms are higher, it might instead mean the online mode of delivery frees up attentional or cognitive resources, but might also indicate potential irregularities or cheating (Steger et al., 2020). In ACE Kids, we instruct parents against undue intervention during parent tutorials, and offer clear guidance about proper conduct, such as not using calculators during arithmetic tasks. We also include tasks that are not easy to cheat in through internet searching, such as the number line tasks or phoneme deletion in the phonological awareness task; or tasks where it is less meaningful to cheat, such as rapid naming tasks in which speedy response is emphasized.

Further, differences in assessment performance can arise from uneven access to devices (Mulligan and Ayoub, 2023), internet access (Hampton et al., 2021); socioeconomic disparity (Guo and Wan, 2022; Wolf et al., 2022) and parent assistance (Du et al., 2025). These factors compound upon the design of the assessment itself, and relate to the design of the overall data collection. Researchers might mitigate these inequalities via a hybrid mode of data collection, allowing travel stipends, in-lab data collection, in-person proctored visits with provided devices, and lowering the technical hurdle involved in the assessment, such as a clean and optimized portal consuming less bandwidth, running well on devices with basic specification, and requiring less peripherals to work, as well as providing proctored alternatives for parents. In ACE Kids, we attempt to address these factors via the aforementioned hybrid mode of data collection, and the training of a dedicated team of more than 20 student helpers who visit homes of parents and children in need, and act as proctors and deliverers of necessary digital devices for at-home testing.

While amelioration measures reduce the uneven access to assessment, the effects of varied testing environment can be further examined from the collected data, particularly the metadata. Some metadata can be automatically gleaned from user interactions through the web app, such as browser environment; others would require explicit questioning. In ACE Kids, we record usage metadata in terms of timestamps, browser environment, device and input type, such as desktop vs. laptop vs. tablets; and for handwriting tasks using fingers vs. stylus. This allows the separating of collected data further according to these attributes to subnorm the dataset, which we plan to do by the end of the data collection cycle. This is especially important in a self-directed and largely unproctored online assessment context, in order to understand the interactions between how participants interface with the assessment platform affects data quality and validity.

### 6.4 Usage rights

When assessments are digitized online, researchers need to be mindful of usage rights that might limit adaptation of paper-and-pencil assessments into online forms. The rights owners of certain psychoeducational assessments, like the Wechsler Intelligence Scale for Children (Wechsler and Kodama, 1949), limit researchers to deliver said assessments in paper-and-pencil formats. Telepractice rules (e.g., Pearson Clinical Assessment UK, 2024) constrain researchers from adapting materials in an online format, or deliver them through teleconferencing. In these cases, researchers can either ask for dispensation from the rights owner, look for or develop equivalent measures, or to arrange for part of the assessments to be delivered in-person.

## 7 Lessons learnt from ACE kids

The article discusses what researchers need to consider when planning and developing a customized online assessment platform for children. To this end, we summarize the following five recommendations: First, before starting, researchers should clarify their project goals, to see if a customized assessment platform offers what they need, or if existing solutions are sufficient. If the goal is to conduct concurrent and multimodal assessments, and there is a need for real-time data processing and integration, then a customized platform is suitable. Second, researchers should plan the scope and features ahead, preferably with experienced personnel in research, project planning and programming, for an estimate of time and resources needed. Third, researchers should expect time and manpower investment and the need for a wide range of programming and design expertise. Fourth, researchers should expect new challenges on top of those associated with the development of paper-and-pencil assessments, such as programming, testing, running costs, and additional validation. Finally, researchers should plan for continued development, including bug fixing, updates, and future uses. The unique challenges in ACE Kids in combining biliteracy assessments, larger and complex assessment battery, longer total testing hours, and usage metadata collection exemplify the need to carefully consider these five points ahead and during assessment building and usage.

By elucidating the issues and challenges that we have identified during the development of the ACE Kids platform, we hope researchers, clinicians and educators can build reliable and scalable online child assessments of their own that fit their diverse and specific needs.

## Data Availability

The deidentified raw data supporting the conclusions of this article will be made available by the authors, without undue reservation.
