# Supplementary material for: Creating online cognitive assessment platform for children’s literacy and math skills: a case study illustrated with the ACE kids platform
Source: Front Hum Neurosci. 2026 Oct 8;20:1882555. doi: 10.3389/fnhum.2026.1882555 (PMC13686991; doi:10.3389/fnhum.2026.1882555)
Supplement: Supplementary file 1 [file Data_Sheet_1.docx]

**Appendix A**

**List of assessments included in the ACE Kids platform (alphabetically ordered)**

| **Child Assessments** | **Remarks** |
| --- | --- |
| Arithmetic Skill | 26 arithmetic items of addition, subtraction, multiplication and division of increasing difficulty. For grade K, arithmetic task is replaced by the addition task, with 9 addition items and numeric comparison task, with 8 items of selecting the larger of the two presented numbers. (Cheung & McBride-Chang, 2015) |
| (For Grade K) Addition |  |
| (For Grade K) Numeric Comparison |  |
| Backward Digit Span | 16 items of 8 levels, each level with 2 trials of the same span length, starting with 2 digits, each trial consists of a random sequence of one-syllable Cantonese digits read out aloud at the beginning of the trial, and the participants read the digits in reverse order. (Ho et al., 2019) |
| Child Questionnaire: Chinese Reading anxiety | An analogous version self-developed based on the English Reading Anxiety scale |
| Child Questionnaire: Chinese Reading motivation | Chinese Reading Motivation Scale adopted from Becker et al. (2010); Lin et al. (2012); Yeung et al. (2020); Yuet-Han Lau and McBride-Chang (2005) |
| Child Questionnaire: English reading anxiety | Foreign Language Reading Anxiety scale translated into Chinese from Saito et al. (1999) |
| Child Questionnaire: English reading motivation | An analogous version self-developed based on the Chinese Reading Motivation scale |
| Child Questionnaire: General child anxiety | Chinese-translated version of Spence Children’s Anxiety Scale (SCAS) Ahlen et al. (2018) |
| Child Questionnaire: Math anxiety | Contain the 5-item scale from OECD as math difficulty factor and adapted a 6-item based on FLRA as math confidence and enjoyment factor. Hamada and Takaki (2021); Rozgonjuk et al. (2020) |
| Child Questionnaire: Math learning motivation | An analogous version self-developed based on the Chinese Reading Motivation scale |
| Child Questionnaire: Medium of Instruction and Preference Scale | Self-developed scale tapping on the preferences of medium of instruction (Chinese vs. English) during other lessons (e.g. mathematics) and daily communications |
| Chinese Delayed Copying | Delayed handwritten copying of 15 single character that are each shown for 2 second (Lo et al., 2018) |
| Chinese Digit Rapid Naming | 1 practice and 2 experimental trials of rapid automatized naming of eight rows of five digits modeled after Denckla and Rudel (1974) |
| Chinese Lexical Decision | 24 rare characters and 24 noncharacters comprising of two components in left-right structure are shown and judged to be a valid Chinese character structure or not. (Huo et al., 2022) |
| Chinese Morphofun | 29 items, in which a picture of an object representing a two-character compound word is shown on each trial, and from eight alternatives, two elements are chosen to form the target word; participants are also asked to identify if the target word represents a real-life object or a creative (does not exist in real life) object.(Shin et al., 2026) |
| Chinese Morphological Awareness | 46 items of construction of novel compound words to describe the object or concept based on known morphemes, adopted from McBride-Chang et al. (2003) |
| Chinese One-Minute Word Reading | One-minute reading aloud as many of 80 two-character words taken from Huo et al. (2022) |
| Chinese Phonological Awareness | 9 syllable deletion and 9 onset deletion items adapted from Tong et al. (2009) |
| Chinese Reading Comprehension | 4 reading passages with 17 multiple-choice questions and 12 open-end questions in total, adapted from Ho et al. (2017) with an additional fourth passage added from the original three. |
| Chinese Single Character Reading | One-character word reading of 160 items of increasing difficulty. Initially adopted from Ho et al. (2017) but new items are drawn from the GRF dataset (Ruan et al., 2024) |
| Chinese Vocabulary Knowledge (definition) | Vocabulary knowledge battery contains 21 receptive items in which children choose the picture from 4 alternatives that best matched the meaning of the word presented; 23 expressive items in which children are asked to name the picture of objects or situation shown; 15 definition items in which children give definitions of words visually presented on the screen. For detailed design refer to Pan et al. (2023) and McBride-Chang et al. (2008) |
| Chinese Vocabulary Knowledge (expressive) |  |
| Chinese Vocabulary Knowledge (receptive) |  |
| Chinese Word Dictation | 20 two-character words are handwritten after audio readout at the beginning of the trial. (Tong et al., 2009) |
| Chinese Word Reading | Two-character word reading of 83 items of increasing difficulty. Initially adopted from Ho et al. (2000) but new items are drawn from the GRF dataset (Ruan et al., 2024) |
| Discourse Skill | 11 test items and 1 practice item of rearrangement of 3-5 sentences to form a meaningful discourse. (Chik et al., 2012; Ho et al., 2017) |
| English Comprehension | 12 items in the Picture Comprehension task where 12 short English sentences are each paired with five picture choices, and participants choose the picture best expressing the meaning of the sentence; 20 items (14 multiple-choice items and 6 long questions) in the Passage Comprehension task from two short English essays. (Li et al., 2012) |
| English Delayed Copying | Similar to Chinese Delayed Copying, except there are delayed handwritten copying of 10 items of low-frequency real words of 6-11 letters (Lo et al., 2018) |
| English Digit Rapid Naming | 1 practice and 2 experimental trials of rapid automatized naming of eight rows of five digits modeled after Denckla et al. (1974) |
| English Invented Spelling | 12 pseudowords of 3-6 letters are read aloud, and the participants type out the spelling they heard with device keyboard (McBride-Chang & Ho, 2005b) |
| English Morphofun | Analogous task design with Chinese Morphofun except with 30 items. (Shin et al., 2026) |
| English Morphological Awareness | 11 items of morpheme compounding (McBride-Chang et al., 2005a), in which children are presented with an example for English compound word and then they were required to create a new word based on the example given; 4 items of WUG test (Berko, 1958) involving inflectional morphemes of making words plural or changing tense, presented with pictures; 5 items of compounding production (Liu & McBride-Chang, 2010), in which children are asked to make up a word in English this time. For example, “What do we call a monster that only eats pizza?” The model answer was pizzaeating monster. |
| English One-Minute Word Reading | Analogous number of items and task design with Chinese One-Minute Word Reading (Huo et al., 2022) |
| English Vocabulary Knowledge (definition) | Analogous trial number and task design with Chinese Vocabulary Knowledge tasks (Tong & McBride-Chang, 2010; Tong et al., 2017) |
| English Vocabulary Knowledge (expressive) |  |
| English Vocabulary Knowledge (receptive) |  |
| English Word Dictation | 12 non-compound words and 18 compound words are handwritten after audio readout at the beginning of the trial. (Lo et al., 2018) |
| English Word Reading | 60 English words arranged in 6 levels of 10 items according to increasing difficulties, an additional level added while adapting from Tong et al. (2010) |
| Number Line | 40 items of 4 ranges (0-10, 0-100, 0-1000, 0-10000) of 10 items, each item with a number line marked with the lowest and highest values of the ranges, and participants moves the toggle on the number line to mark the position of the target number on the line. (Chan & Wong, 2016; Siegler & Opfer, 2003) |
| Silent Word Reading | 25 items in individual silent English word reading in which participants demarcate (via clicking on the word boundary) words formed from equidistant letters; 20 sentences without space between words are also similarly presented in silent contextual reading (Kalindi et al., 2015a; Kalindi et al., 2015b) |
| Strategic Counting | Prior to the formal test, the experimenter explained the equivalence between four quantities by illustration. Small squares, ten bars, hundred squares and thousand cubes were presented on a sheet of paper to the children. A ten bar contains 10 small squares. A hundred square contains 10 bars (i.e., 100 small squares). A thousand cube contains 10 hundred squares (i.e., 1000 small squares).  16 items of 3 sets; 5 items of two-digit quantities, 5 items of three-digit quantities and 6 items of four-digit quantities were displayed, and the children type the number to represent the quantities of the small square. (Chan et al., 2014; Wong, 2019) |
| Word Problem | 31 word problem questions assessing whole number knowledge, fractions and decimal framed as money problem, time problem and problem on comparisons; adopted with revision from the Learning Achievement Measurement Kit (LAMK), a local mathematics achievement test standardized by the Hong Kong Education Bureau (Education Bureau, 2008) |

| **Parent Assessments** | **Remarks** |
| --- | --- |
| Parent Questionnaire: Attitudes Towards Childhood Multilingualism | Scale adapted and translated into Chinese from Kircher et al. (2022) |
| Parent Questionnaire: Background information | Self-developed scale for basic demographical information, learning difficulties and SES. |
| Parent Questionnaire: Child Executive Functioning Inventory | Childhood executive functions inventory (CHEXI) adapted and translated into Chinese from Catale et al. (2015) |
| Parent Questionnaire: Home literacy practices Inventory (Chinese, English, Bilingual) | Home reading and literacy practice scales adapted and translated from Georgiou et al. (2021); Gonzalez-Barrero et al. (2021) |
| Parent Questionnaire: Home numeracy inventory | Home Numeracy Practice scale adapted from Wei et al. (2022) |
| Parent Questionnaire: Parents’ expectations on learning Chinese and English | Self-developed scale for parental views on the importance and attitude over children’s learning Chinese and English. |
| Parent Questionnaire: Reading interests in Chinese and English | Reading interest scale in Chinese and English adapted from Chow et al. (2011) |
| Parent Testing: Arithmetic Skill | Same trial structure and task design as child measure. |
| Parent Testing: Chinese One-Minute Word Reading | Same task design as child measure, 3 trials consisting of consistent characters, inconsistent characters and two-character word reading respectively (Siu et al., 2018) |
| Parent Testing: Chinese Word Dictation | Same task design as child measure with 10 new items created for parent testing. |
| Parent Testing: Silent Word Reading | Same trial structure and task design as child measure. |

**Appendix B**

**Technical Specifications of the ACE Kids E-platform**

| **Tech Stack** | Backend: Flask version 2.0.3, Python 3.6 Frontend: jsPsych 6.3 |
| --- | --- |
| **Hosting Environment** | On-Premise Ubuntu 18.04.5 LTS |
| **Server Gateway** | HTTPS to Nginx Reverse Proxy to uWSGI to Flask web app |
| **Deployment Workflow** | Git separated into frontend and backend components, with master branch and development branches on individual features |
| **Database Engine** | MariaDB 10.3.28, index variables as VARCHAR and data stored as text blobs |
| **Core Tables** | Users, Session Data (logging in and out), Usage Log (logging access of task and trial data upload), Individual Task Data Tables (Anonymized ID, trial index and data, trial timestamp).  Relations are kept using persistent anonymized subject IDs across tables. |
| **Protocol** | Authentication via Username/Password Session managed server-side via Flask-Session |
| **Password Security** | Hashed with Werkzerg 2.0.2’s pbkdf2:sha256 |
| **Encryption** | TLS v1.2, AES-256-GCM for PII data stored at rest |
| **Web Protection** | Explicit PyMySQL statements to prevent SQL injections, with additional SQLAlchemy wrapper |
| **Backup Protocol** | Daily automatic snapshots, database backup separately saved onto on-premise NAS drive |

**Appendix D**

**Cognitive Load and Extraneous Cognitive Load Questionnaire Template**

**Part 1:** 認知負荷 (**Cognitive load)**

Instruction: Please select the most suitable option for you from 1-9

1. In the task I just finished, I have used ……

*Mental effort refers to how much work on your mind you paid to the task.

|  | Very, very low mental effort | Very low mental effort | Low mental effort | Rather low mental effort | Neither low or high mental effort | Rather high mental effort | High mental effort | Very high mental effort | Very, very high mental effort |
| --- | --- | --- | --- | --- | --- | --- | --- | --- | --- |
| Test 1 | 1 | 2 | 3 | 4 | 5 | 6 | 7 | 8 | 9 |
| Test 2 | 1 | 2 | 3 | 4 | 5 | 6 | 7 | 8 | 9 |
| Test 3 | 1 | 2 | 3 | 4 | 5 | 6 | 7 | 8 | 9 |
| Test 4  （If any） | 1 | 2 | 3 | 4 | 5 | 6 | 7 | 8 | 9 |
| Test 5  （If any） | 1 | 2 | 3 | 4 | 5 | 6 | 7 | 8 | 9 |

**Part 2:** 外在認知負荷 (**Extraneous cognitive load)**

Instruction: Please select the most suitable option for you from 1-9

|  | | Very, very easy | Very easy | Easy | Rather easy | Neither easy or difficult | Rather difficult | Difficult | Very difficult | Very, very difficult |
| --- | --- | --- | --- | --- | --- | --- | --- | --- | --- | --- |
| 1a | Using a screen to read | 1 | 2 | 3 | 4 | 5 | 6 | 7 | 8 | 9 |
| 1b | Using a mouse to answer questions | 1 | 2 | 3 | 4 | 5 | 6 | 7 | 8 | 9 |

Instruction: Please select the most suitable option for you from 1-9

|  | | Completely disagree  1 | 2 | 3 | 4 | Neither agree or disagree  5 | 6 | 7 | 8 | Completely agree  9 |
| --- | --- | --- | --- | --- | --- | --- | --- | --- | --- | --- |
| 2 | The **word** instructions during the test were very unclear. | 1 | 2 | 3 | 4 | 5 | **6** | **7** | **8** | **9** |
| 3 | The **audio** instructions during the test were very unclear. | 1 | 2 | 3 | 4 | 5 | **6** | **7** | **8** | **9** |
| 4 | The **word** instructions were very effective. | **1** | **2** | **3** | **4** | 5 | 6 | 7 | 8 | 9 |
| 5 | The **audio** instructions were very effective. | **1** | **2** | **3** | **4** | 5 | 6 | 7 | 8 | 9 |
| 6 | The **interface** of the test is very easy to use. | **1** | **2** | **3** | **4** | 5 | 6 | 7 | 8 | 9 |

*If a participant answers "agree" (6-9) for questions 2 and 3, or "disagree" (1-5) for questions 4, 5, and 6, please ask for detailed reasons and record them on the next page.*

**Part 3: 使用的電子設備 (The digital device used)**

1. Which of the following electronic devices did the participant use to conduct the test?

| 1 | 2 | 3 | 4 |
| --- | --- | --- | --- |
| Desktop computer with mouse | Tablet with mouse | Laptop with touchpad | Other (Please specify: _____) |

1. What operating system did the electronic device used by the participant use?

| 1 | 2 | 3 |
| --- | --- | --- |
| MacOS | Windows | Other (Please specify: _____) |

1. What electronic device did the participant use most frequently?

| 1 | 2 | 3 | 4 |
| --- | --- | --- | --- |
| Desktop computer | Laptop | Tablet | Other (Please specify: _____) |

**Part 4: 顯示屏尺寸 (The size of the display screen)**

The display area of the reading device refers to the length of its diagonal (in centimeters). Please measure the distance between the diagonals of the display area. The following is a demonstration of the measurement:

The diagonal of the participant's reading device display screen is _________________ centimeters.

**References**

Ahlen, J., Vigerland, S., & Ghaderi, A. (2018). Development of the spence children’s anxiety scale-short version (SCAS-S). *Journal of psychopathology and behavioral assessment, 40*(2), 288–304. <https://doi.org/10.1007/s10862-017-9637-3>

Becker, M., McElvany, N., & Kortenbruck, M. (2010, Nov). Intrinsic and extrinsic reading motivation as predictors of reading literacy: A longitudinal study. *Journal of Educational Psychology, 102*(4), 773. <https://doi.org/10.1037/a0020084>

Berko, J. (1958). The child's learning of English morphology. *Word, 14*(2-3), 150–177. <https://doi.org/10.1080/00437956.1958.11659661>

Catale, C., Meulemans, T., & Thorell, L. B. (2015, Jun). The childhood executive function inventory: Confirmatory factor analyses and cross-cultural clinical validity in a sample of 8-to 11-year-old children. *Journal of attention disorders, 19*(6), 489–495. <https://doi.org/10.1177/1087054712470971>

Chan, W. W. L., Au, T. K., & Tang, J. (2014, Feb). Strategic counting: A novel assessment of place-value understanding. *Learning and instruction, 29*, 78–94. <https://doi.org/10.1016/j.learninstruc.2013.09.001>

Chan, W. W. L., & Wong, T. T.-Y. (2016, Aug). The underlying number–space mapping among kindergarteners and its relation with early numerical abilities. *Journal of Experimental Child Psychology, 148*, 35–50. <https://doi.org/10.1016/j.jecp.2016.03.010>

Cheung, S. K., & McBride-Chang, C. (2015). Evaluation of a Parent Training Program for Promoting Filipino Young Childrens Number Sense with Number Card Games. *Child Studies in Asia-Pacific Contexts, 5*(1), 39–49. <https://doi.org/10.5723/csac.2015.5.1.039>

Chik, P. P.-m., Ho, C. S.-h., Yeung, P.-s., Wong, Y.-k., Chan, D. W.-o., Chung, K. K.-h., & Lo, L.-y. (2012, Apr). Contribution of discourse and morphosyntax skills to reading comprehension in Chinese dyslexic and typically developing children. *Annals of Dyslexia, 62*(1), 1–18. <https://doi.org/10.1007/s11881-010-0045-6>

Chow, B. W., Ho, C. S., Wong, S. W., Waye, M. M., & Bishop, D. V. (2011, Feb 10). Genetic and environmental influences on Chinese language and reading abilities. *PLoS ONE, 6*(2), e16640. <https://doi.org/10.1371/journal.pone.0016640>

Clinton, V. (2019, May). Reading from paper compared to screens: A systematic review and meta‐analysis. *Journal of Research in Reading, 42*(2), 288–325. <https://doi.org/10.1111/1467-9817.12269>

Cottin, M., Blum, K., Konjufca, J., Quevedo, Y., Kaaya, S., Behn, A., Schmeck, K., Sharp, C., & Zimmermann, R. (2022, Jun 3). Digital use of standardised assessment tools for children and adolescents: can available paper-based questionnaires be used free of charge in electronic format? *BMC psychiatry, 22*(1), 379. <https://doi.org/10.1186/s12888-022-04023-w>

Denckla, M. B., & Rudel, R. G. (1974). Rapid "automatized" naming (R.A.N.): Dyslexia differentiated from other learning disabilities. *Cortex, 14*, 471–479. <https://doi.org/10.1016/0028-3932(76)90075-0>

Education Bureau. (2008). *Learning achievement measurement Kit 2.0: User’s guide.* Education Bureau.

Georgiou, G. K., Inoue, T., & Parrila, R. (2021, Sep). Developmental relations between home literacy environment, reading interest, and reading skills: Evidence from a 3-year longitudinal study. *Child Development, 92*(5), 2053–2068. <https://doi.org/10.1111/cdev.13589>

Gonzalez-Barrero, A. M., Salama-Siroishka, N., Dubé, D., Brouillard, M., & Byers-Heinlein, K. (2021). Effects of language dominance on home reading practices of bilingual families. *International Journal of Bilingualism, 25*(1), 77–99. <https://doi.org/10.1177/1367006920938153>

Gustafsson, H. C., Young, A. S., Stamos, G., Wilken, S., Brito, N. H., Thomason, M. E., Graham, A., Nigg, J. T., & Sullivan, E. L. (2021, Dec). Innovative methods for remote assessment of neurobehavioral development. *Developmental cognitive neuroscience, 52*, 101015. <https://doi.org/10.1016/j.dcn.2021.101015>

Hamada, A., & Takaki, S. (2021, Oct). Approximate replication of Matsuda and Gobel (2004) for psychometric validation of the Foreign Language Reading Anxiety Scale. *Language Teaching, 54*(4), 535–551. <https://doi.org/10.1017/S0261444819000296>

Heiervang, E., & Goodman, R. (2011, Jan). Advantages and limitations of web-based surveys: evidence from a child mental health survey. *Social psychiatry and psychiatric epidemiology, 46*(1), 69–76. <https://doi.org/10.1007/s00127-009-0171-9>

Ho, C. S.-H., Chan, D. W.-O., Tsang, S. M. A., & Lee, S. H. (2000). The Hong Kong test of specific learning disabilities in reading and writing (HKT-SpLD). *Hong Kong, China: Chinese University of Hong Kong and Education Department, HKSAR Government*.

Ho, C. S.-H., Fong, C. Y.-C., & Zheng, M. (2019, Mar). Contributions of vocabulary and discourse-level skills to reading comprehension among Chinese elementary school children. *Applied Psycholinguistics, 40*(2), 323–349. <https://doi.org/10.1017/S0142716418000590>

Ho, C. S.-H., Zheng, M., McBride, C., Hsu, L. S. J., Waye, M. M., & Kwok, J. C.-Y. (2017, Oct). Examining an extended simple view of reading in Chinese: The role of naming efficiency for reading comprehension. *Contemporary Educational Psychology, 51*, 293–302. <https://doi.org/10.1016/j.cedpsych.2017.08.009>

Huo, S., Wu, K. C., Mo, J., Wang, J., & Maurer, U. (2022, May–Jun). Children with Chinese dyslexia acquiring English literacy: Interaction between cognitive subtypes of dyslexia and orthographies. *Journal of Learning Disabilities, 55*(3), 229–241. <https://doi.org/10.1177/00222194211017819>

Kalindi, S. C., McBride, C., Chan, S., Chung, K. H. K., Lee, C.-Y., Maurer, U., & Tong, X. (2015a). A short test of English silent word reading for English language learners. *Child Studies in Asia-Pacific Contexts, 5*(2), 95–105. <https://doi.org/10.5723/csac.2015.5.2.095>

Kalindi, S. C., McBride, C., Tong, X., Wong, N. L. Y., Chung, K. H. K., & Lee, C.-Y. (2015b, Jul). Beyond phonological and morphological processing: Pure copying as a marker of dyslexia in Chinese but not poor reading of English. *Annals of Dyslexia, 65*(2), 53–68. <https://doi.org/10.1007/s11881-015-0097-8>

Kircher, R., Quirk, E., Brouillard, M., Ahooja, A., Ballinger, S., Polka, L., & Byers-Heinlein, K. (2022, Oct). Quebec-based parents’ attitudes towards childhood multilingualism: Evaluative dimensions and potential predictors. *Journal of Language and Social Psychology, 41*(5), 527–552. <https://doi.org/10.1177/0261927X221078853>

Li, T., McBride-Chang, C., Wong, A., & Shu, H. (2012, May). Longitudinal predictors of spelling and reading comprehension in Chinese as an L1 and English as an L2 in Hong Kong Chinese children. *Journal of Educational Psychology, 104*(2), 286. <https://doi.org/10.1037/a0026445>

Lin, D., Wong, K. K., & McBride-Chang, C. (2012, Mar). Reading motivation and reading comprehension in Chinese and English among bilingual students. *Reading and Writing, 25*(3), 717–737. <https://doi.org/10.1007/s11145-011-9297-8>

Liu, P. D., & McBride-Chang, C. (2010). What is morphological awareness? Tapping lexical compounding awareness in Chinese third graders. *Journal of Educational Psychology, 102*(1), 62–73. <https://doi.org/10.1037/a0016933>

Lo, J. C. M., Ye, Y., Tong, X., McBride, C., Ho, C. S.-h., & Waye, M. M. Y. (2018). Delayed copying is uniquely related to dictation in bilingual Cantonese–English-speaking children in Hong Kong. *Writing Systems Research, 10*(1), 26–42. <https://doi.org/10.1080/17586801.2018.1481902>

McBride-Chang, C., Cho, J.-R., Liu, H., Wagner, R. K., Shu, H., Zhou, A., Cheuk, C. S., & Muse, A. (2005a, Oct). Changing models across cultures: Associations of phonological awareness and morphological structure awareness with vocabulary and word recognition in second graders from Beijing, Hong Kong, Korea, and the United States. *Journal of Experimental Child Psychology, 92*(2), 140–160. <https://doi.org/10.1016/j.jecp.2005.03.009>

McBride-Chang, C., & Ho, C. S.-H. (2005b). Predictors of beginning reading in Chinese and English: A 2-year longitudinal study of Chinese kindergartners. *Scientific Studies of Reading, 9*(2), 117–144. <https://doi.org/10.1207/s1532799xssr0902>

McBride-Chang, C., Shu, H., Zhou, A., Wat, C. P., & Wagner, R. K. (2003, Dec). Morphological awareness uniquely predicts young children's Chinese character recognition. *Journal of Educational Psychology, 95*(4), 743–751. <https://doi.org/10.1037/0022-0663.95.4.743>

McBride-Chang, C., Tardif, T., Cho, J.-R., Shu, H., Fletcher, P., Stokes, S. F., Wong, A., & Leung, K. (2008). What's in a word? Morphological awareness and vocabulary knowledge in three languages. *Applied Psycholinguistics, 29*(3), 437–462. <https://doi.org/10.1017/S014271640808020X>

Pan, D. J., Nakayama, M., McBride, C., Cheah, Z. R. E., Zheng, M., & Yeung, C. C. L. (2023, Jan). Cognitive-linguistic skills and vocabulary knowledge breadth and depth in children’s L1 Chinese and L2 English. *Applied Psycholinguistics, 44*(1), 77–99. <https://doi.org/10.1017/S0142716422000480>

Pramana, G., Parmanto, B., Lomas, J., Lindhiem, O., Kendall, P. C., & Silk, J. (2018, May 10). Using mobile health gamification to facilitate cognitive behavioral therapy skills practice in child anxiety treatment: open clinical trial. *JMIR serious games, 6*(2), e8902. <https://doi.org/10.2196/games.8902>

Rozgonjuk, D., Kraav, T., Mikkor, K., Orav-Puurand, K., & Täht, K. (2020, Sep 7). Mathematics anxiety among STEM and social sciences students: the roles of mathematics self-efficacy, and deep and surface approach to learning. *International journal of STEM education, 7*(1), 46. <https://doi.org/10.1186/s40594-020-00246-z>

Ruan, Y., Ye, Y., & McBride, C. (2024). Effectiveness of parent coaching on the literacy skills of Hong Kong Chinese Children with and without dyslexia. *Reading and Writing, 37*(7), 1805–1826. <https://doi.org/10.1007/s11145-022-10408-w>

Saito, Y., Garza, T. J., & Horwitz, E. K. (1999, Sum). Foreign language reading anxiety. *The modern language journal, 83*(2), 202–218. <https://doi.org/10.1111/0026-7902.00016>

Salmerón, L., Delgado, P., Vargas, C., & Gil, L. (2021). Tablets for all? Testing the screen inferiority effect with upper primary school students. *Learning and Individual Differences, 86*, 101975. <https://doi.org/10.1016/j.lindif.2021.101975>

Shields, M. M., McGinnis, M. N., & Selmeczy, D. (2021). Remote research methods: Considerations for work with children. *Frontiers in Psychology, 12*, 703706. <https://doi.org/10.3389/fpsyg.2021.703706>

Shin, H., Ariza, J. D., Cheah, Z. R. E., Ng, M. C. Y., Ngan, V. S. H., Mo, J., Schumaker, J., Marks, R. A., Maurer, U., & McBride, C. A. (2026, Mar 14). Unique predictors of cognitive-linguistic skills in English word reading through online assessment. *Reading and Writing*, 1–22. <https://doi.org/10.1007/s11145-026-10772-x>

Siegler, R. S., & Opfer, J. E. (2003, May). The development of numerical estimation: Evidence for multiple representations of numerical quantity. *Psychological Science, 14*(3), 237–250. <https://doi.org/10.1111/1467-9280.02438>

Siu, T.-S. C., McBride, C., Tse, C.-S., Tong, X., & Maurer, U. (2018). Evaluating the effects of metalinguistic and working memory training on reading fluency in Chinese and English: A randomized controlled trial. *Frontiers in Psychology, 9*, 2510. <https://doi.org/10.3389/fpsyg.2018.02510>

Tabachnick, A. R., Sellers, T., Margolis, E., Labella, M., Neff, D., Crowell, S., Raby, K. L., Saenz, C., Conradt, E., & Dozier, M. (2022). Adapting psychophysiological data collection for COVID‐19: The “virtual assessment” model. *Infant mental health journal, 43*(1), 185–197. <https://doi.org/10.1002/imhj.21954>

Tandon, S. D., Chavez, J., Diebold, A., Moses, A., Lovejoy, A. E., Wang, Z., Arevalo, K., McBride, E., Brennan, M., & Anderson, E. (2026, Jan). Zoom to the virtual room: The shift to remote early childhood observational assessments. *Prevention Science, 27*(1), 28–40. <https://doi.org/10.1007/s11121-024-01737-z>

Tong, X., & McBride-Chang, C. (2010, Mar). Chinese-English biscriptal reading: Cognitive component skills across orthographies. *Reading and Writing, 23*(3), 293–310. <https://doi.org/10.1007/s11145-009-9211-9>

Tong, X., McBride-Chang, C., Shu, H., & Wong, A. M. Y. (2009). Morphological Awareness, Orthographic Knowledge, and Spelling Errors: Keys to Understanding Early Chinese Literacy Acquisition. *Scientific Studies of Reading, 13*(5), 426–452. <https://doi.org/10.1080/10888430903162910>

Tong, X., McBride, C., Ho, C. S.-h., Waye, M. M. Y., Chung, K. K. H., Wong, S. W. L., & Chow, B. W. Y. (2017). Within- and cross-language contributions of morphological awareness to word reading and vocabulary in Chinese–English bilingual learners. *Reading and Writing, 31*(8), 1765–1786. <https://doi.org/10.1007/s11145-017-9771-z>

Wei, W., Wang, Q.-Y., Luo, Q., & Li, Y. (2022). Cross-lagged relationship between home numeracy practices and early mathematical skills among Chinese young children. *Frontiers in Psychology, 13*, 1033065. <https://doi.org/10.3389/fpsyg.2022.1033065>

Wong, T. T. Y. (2019, Dec). The roles of place‐value understanding and non‐symbolic ratio processing system in symbolic rational number processing. *British Journal of Educational Psychology, 89*(4), 635–652. <https://doi.org/10.1111/bjep.12249>

Yeung, P.-s., Ho, C. S.-h., Chan, D. W.-o., & Chung, K. K.-h. (2020). Writing motivation and performance in Chinese children. *Reading and Writing, 33*(2), 427–449. <https://doi.org/10.1007/s11145-019-09969-0>

Yuet-Han Lau, J., & McBride-Chang, C. (2005). Home literacy and Chinese reading in Hong Kong children. *Early education and development, 16*(1), 5–22. <https://doi.org/10.1207/s15566935eed1601_1>
