# Supplementary material for: Creating online cognitive assessment platform for children’s literacy and math skills: a case study illustrated with the ACE kids platform
Source: Front Hum Neurosci. 2026 Oct 8;20:1882555. doi: 10.3389/fnhum.2026.1882555 (PMC13686991; doi:10.3389/fnhum.2026.1882555)
Supplement: Supplementary file 2 [file Data_Sheet_2.DOCX]

|  | Test Items | Weak | Fair | Average | Good | Excellent |
| --- | --- | --- | --- | --- | --- | --- |
| Chinese | Oral Vocabulary - Tests students' knowledge of Chinese vocabulary and word formation ability.。 |  |  |  |  |  |
|  | Word Recognition - Tests students' accuracy in reading and writing Chinese words. |  |  |  |  |  |
|  | Word Dictation - Tests students' accuracy in dictating Chinese words. |  |  |  |  |  |
|  | Text Comprehension - Tests students' ability to understand the main idea of sentences or paragraphs and infer context. |  |  |  |  |  |
| English | Oral Vocabulary - Tests students' knowledge of English vocabulary and word formation ability. |  |  |  |  |  |
|  | Word Recognition - Tests students' accuracy in reading and writing English words. |  |  |  |  |  |
|  | Word Dictation - Tests students' accuracy in dictating English words. |  |  |  |  |  |
|  | Reading Fluency - Tests students' ability to read English without reading aloud. |  |  |  |  |  |
| Mathematics | Integer Arithmetic - Tests students' ability to perform addition, subtraction, multiplication, division, and mixed arithmetic operations. |  |  |  |  |  |
|  | Numerical Concepts - Tests students' understanding of the decimal system and their accuracy in judging numerical values and distances. |  |  |  |  |  |
|  | Mathematical Application - Tests students' mathematical application and problem-solving abilities. |  |  |  |  |  |

Note: This reflects the student's performance relative to other participants of the same grade in this study.

Student Performance and Learning Suggestions (Example)

The student performed weaker than peers in both English and Chinese tests, but performed best in Chinese text comprehension.

Among different subject areas, the student's English vocabulary memorization ability was the weakest and needs more effort to improve. We suggest parents encourage their children to read words aloud more often, paying attention to the letter-sound relationships, which helps with phonics development. For example, reading aloud words like Pen, Ken, and Ten can help children distinguish the letters 'p', 'k', and 't'. Furthermore, when reading multi-syllabic words, parents can encourage children to break down the words and read them sequentially. For example, panda can be broken down into /pan/ and /da/; beautiful can be broken down into /beau/, /ti/, and /ful/ (Dunn, 2018). The final step in word association is memorization. When words are broken down into smaller groups and accompanied by the pronunciation of syllables, the connection between letters and sounds makes it easier to string the words together. Repeated use of word-splitting techniques gradually improves a child's sense of sound, making word association more efficient; even in unseen dictation, they can roughly string together unfamiliar words by listening to the pronunciation (Wong, 2018). Children can then spell English words more easily, and longer words won't intimidate them, making them less resistant to English dictation.

Meanwhile, students also perform weaker in English reading fluency. English reading fluency depends on familiarity with English vocabulary. We suggest parents play English word-building games with their children (e.g., Scrabble). When parents encounter unfamiliar words, they can explain them, allowing students to review and reinforce their knowledge through play, thus improving their English reading fluency.

The student performed well in integer arithmetic operations. We suggest parents incorporate math games into daily life to give the student more time to think and practice, maintain their motivation to learn math, and further improve their mathematical calculation skills.

Finally, the student's excellent performance in the math test is commendable. However, we feel there is room for improvement in their application of mathematical concepts. To achieve better results in word problems, we suggest they mark keywords in the questions, use visual aids to construct the logical sequence of solutions, and repeat the questions when necessary to effectively reduce careless mistakes. Additionally, more reading can help improve vocabulary comprehension, which also aids in understanding math word problems.

The student performed relatively well in integer arithmetic operations. We suggest challenging them with more difficult math problems or improving their calculation speed to maintain their motivation to learn math and further improve their mathematical calculation skills.

Disclaimer: All data is for reference only and should not be considered as any professional diagnosis or other purpose.
